# SKYSCRAPER-05: A Phase 2 Study of Perioperative Tiragolumab Plus Atezolizumab, With or Without Platinum-Based Chemotherapy, in Patients With Previously Untreated, Locally Advanced, Resectable, Stage II‒IIIB NSCLC

**DOI:** 10.1016/j.jtocrr.2026.101047

**Published:** 2026-07-10

**Authors:** Catherine A. Shu, Anthony W. Kim, Min Hee Hong, Alex Martinez Marti, Martin Früh, Andrea Ferris, Harvey Pass, Bryan A. Chan, Saiama N. Waqar, Coen A. Bernaards, Namrata S. Patil, Jessie J. Hsu, Cristal Parsons, Sarah Troutman, Christina Matheny, Pao-Chen Li, Barbara Gitlitz, Enriqueta Felip

**Affiliations:** a Division of Hematology and Medical Oncology, Department of Medicine, Herbert Irving Comprehensive Cancer Center, Columbia University, New York, New York; b Division of Thoracic Surgery, Department of Surgery, Keck School of Medicine, University of Southern California, Los Angeles, California; c Department of Internal Medicine, Yonsei Cancer Center, Severance Hospital, Yonsei University College of Medicine, Seoul, Republic of Korea; d Department of Medical Oncology, Vall d’Hebron University Hospital, Vall d’Hebron Institute of Oncology, Barcelona, Spain; e Department of Medical Oncology and Haematology, Cantonal Hospital St. Gallen, HOCH Health Ostschweiz, St. Gallen, Switzerland and Department of Medical Oncology, University of Bern, Bern, Switzerland; f LUNGevity Foundation, Chicago, Illinois; g Department of Cardiothoracic Surgery, New York University Langone Health, Grossman School of Medicine, New York University, New York, New York; h Department of Medical Oncology, The Adem Crosby Cancer Centre, Sunshine Coast University Hospital, Birtinya, Queensland, Australia; i Division of Oncology, Siteman Cancer Center, Washington University School of Medicine, St. Louis, Missouri; j Product Development Data Science & Analytics, Genentech, Inc., South San Francisco, California; k Translational Medicine, Genentech, Inc., South San Francisco, California; l Product Development Clinical Safety, Genentech, Inc., South San Francisco, California; m Product Development Oncology, Genentech, Inc., South San Francisco, California; n Department of Medical Oncology, Vall d’Hebron University Hospital, Vall d’Hebron Institute of Oncology, Universitat Autònoma de Barcelona, Barcelona, Spain

**Keywords:** Atezolizumab, Chemotherapy, Immunotherapy, Resectable NSCLC, Tiragolumab

## Abstract

**Introduction:**

The SKYSCRAPER-05 (NCT04832854), a phase 2, open-label, multicenter study, evaluated perioperative treatment with tiragolumab plus atezolizumab ± platinum-based chemotherapy in treatment-naive patients with resectable, stage II−IIIB NSCLC.

**Methods:**

Cohort A (programmed death ligand-1 [PD-L1]-high) received 4 cycles of neoadjuvant tiragolumab (600 mg IV) plus atezolizumab (1200 mg IV) every 3 weeks, surgery, and the investigator’s choice of adjuvant tiragolumab plus atezolizumab (16 cycles) or platinum-based chemotherapy (4 cycles). Cohort B (PD-L1 all-comers) received tiragolumab plus atezolizumab plus platinum-based chemotherapy (4 cycles), surgery, and tiragolumab plus atezolizumab every 3 weeks (16 cycles). The co-primary end points were major pathologic response (MPR), surgical feasibility, and safety; the secondary end points were pathologic complete response and event-free survival.

**Results:**

At clinical cutoff (February 14, 2024), 50 patients were enrolled (70% male; 66% White), and 48 received treatment (Cohort A, n = 7; Cohort B, n = 41; median follow-up, 9.5 months); 96% of whom underwent resection (4% pneumonectomy, 91% lobectomy or bilobectomy; 93% R0). Cohort A was halted early because of changing treatment paradigms. Cohort B MPR was 51% (95% confidence interval: 35–67) and pathologic complete response was 29% (95% confidence interval: 17–46). Event-free survival was immature. At safety cutoff (March 05, 2025), grade 3 or higher treatment-related adverse events (AEs) occurred in 29% and 44% of patients in Cohorts A and B, respectively; surgery-related grade 3 or higher AEs occurred in 14% and 5%. AEs led to surgical delays in 2 patients and treatment discontinuation in 14 patients (34.1%, all in Cohort B).

**Conclusions:**

Perioperative tiragolumab combined with atezolizumab ± chemotherapy proved to be surgically feasible, with acceptable MPR rates and a safety profile aligned with expectations for resectable, locally advanced NSCLC.

## Introduction

In early-stage, resectable NSCLC, several phase 3 clinical trials have reported the benefit of immune checkpoint inhibitors (ICIs), such as anti-programmed death-ligand 1 (PD-L1) or anti-programmed death-1 (PD-1) antibodies. This benefit has been shown with 2 main strategies: ICIs as monotherapy following chemotherapy in the adjuvant setting, and ICIs in combination with chemotherapy in the perioperative setting.^1^, ^2^, ^3^, ^4^, ^5^ Collectively, these trials established the use of ICIs and chemotherapy in either the neoadjuvant, adjuvant, or perioperative settings as the current standard of care for NSCLC without actionable mutations. Despite these advances, the full potential of immunotherapy in the perioperative setting is still being explored. Novel combinations that enhance antitumor immunity while maintaining a favorable safety profile remain a vital area of investigation to further reduce recurrence rates and improve the cure potential for these patients.

TIGIT (T-cell immunoreceptor with immunoglobulin and ITIM domain) is an immune checkpoint that is highly expressed on natural killer cells, CD4+ and CD8+ T cells, and regulatory T cells.^6^^,^^7^ TIGIT binds with high affinity to the polio virus receptor, preventing the co-stimulatory CD226 ligand from activating T cells and natural killer cells. PD-1 also negatively regulates CD226 activity.^8^^,^^9^ Over 45% of lung cancers show high TIGIT expression, which correlates with PD-1 expression levels, particularly in tumor-infiltrating lymphocytes.^6^^,^^10^

Tiragolumab is an Fc-active human monoclonal antibody that blocks TIGIT-mediated immunosuppression and may augment antitumor responses. Tiragolumab acts on immunosuppressive immune cells in the tumor microenvironment when it is combined with PD-L1/PD-1 checkpoint inhibitors, such as the anti-PD-L1 antibody atezolizumab.^11^ Potentially synergistic antitumor activity for tiragolumab combined with atezolizumab was observed in patients with recurrent or metastatic NSCLC in the phase 1 GO30103 and phase 2 CITYSCAPE studies.^12^^,^^13^ Activity increased with higher PD-L1 expression, resulting in a clinically meaningful reduction in the risk of disease progression or death with this combination compared with atezolizumab alone.^13^

Several other factors provide further rationale for investigating the addition of tiragolumab to atezolizumab-based therapy for NSCLC in earlier treatment settings, including the established role of atezolizumab monotherapy as an adjuvant treatment for PD-L1-positive, resectable NSCLC and its efficacy alone or in combination with chemotherapy in the neoadjuvant setting.^5^^,^^14^^,^^15^ We conducted the phase 2 SKYSCRAPER-05 study (NCT04832854) to investigate the safety and efficacy of this combination in the perioperative setting. Based on the treatment landscape at the time the study was designed, 2 treatment arms were included: one evaluating tiragolumab plus atezolizumab alone in patients whose tumors had high expression of PD-L1 (≥50% tumor cells [TC]) and another evaluating tiragolumab plus atezolizumab plus platinum-based chemotherapy, irrespective of PD-L1 expression level.

## Materials and Methods

### Study Design and Patients

SKYSCRAPER-05 was a phase 2, open-label, multicenter study of perioperative tiragolumab plus atezolizumab, with or without platinum-based chemotherapy, in patients with previously untreated, locally advanced, resectable stage II‒IIIB NSCLC. Patients were enrolled at 20 sites across 5 countries.

Eligible patients were aged 18 years or older, with histologically or cytologically confirmed stage II, IIIA, or select IIIB (T3N2 only) NSCLC per the American Joint Committee on Cancer (AJCC) 8th edition,^16^ and were eligible to receive platinum-based chemotherapy. Tumor samples were required for determination of PD-L1 status, which was assessed using the VENTANA PD-L1 (SP263) immunohistochemistry assay (VENTANA Benchmark Ultra, Tucson, AZ) via central testing. Patients needed adequate pulmonary function to be eligible for R0 surgical resection with curative intent, measurable disease per Response Evaluation Criteria in Solid Tumors v1.1, and an Eastern Cooperative Oncology Group performance status of 0 or 1. Patients with NSCLC with an activating *EGFR* mutation or anaplastic lymphoma kinase (*ALK*) fusion oncogene or known *ROS1* rearrangement, active or a history of autoimmune disease or immune deficiency, or significant cardiovascular disease were excluded. Prior treatment for lung cancer was not permitted.

The study was conducted in accordance with the Declaration of Helsinki and Good Clinical Practice Guidelines. Written informed consent was obtained from all patients before any study-related procedures. The study protocol was approved by the relevant local institutional review board at each study site.

### Procedures

Patients were assigned to one of 2 cohorts: Cohort A included patients whose tumors were defined as PD-L1 high (PD-L1 TC expression levels ≥50% via central testing per the investigational Ventana PD-L1 [SP263] CDx assay [Roche Diagnostics, Indianapolis. IN, USA]); whereas Cohort B included PD-L1 all-comers (i.e., patients whose tumors had any PD-L1 expression level). In Cohort A, patients received 4 cycles of neoadjuvant tiragolumab (600 mg by intravenous [IV] infusion on Day 1 of each cycle) plus atezolizumab (1200 mg IV on Day 1 of each cycle) every 3 weeks (Q3W), followed by surgical resection and then the investigator’s choice of either 16 cycles of adjuvant tiragolumab plus atezolizumab Q3W or 4 cycles of adjuvant platinum-doublet chemotherapy. In Cohort B, patients received 4 cycles of neoadjuvant tiragolumab (600 mg IV on Day 1 of each cycle) plus atezolizumab (1200 mg IV on Day 1 of each cycle) Q3W, plus platinum-doublet chemotherapy, followed by surgical resection and then 16 cycles of adjuvant tiragolumab plus atezolizumab Q3W. Postoperative radiotherapy was optional per local guidelines if residual disease or pathological N2-positive disease was observed at the time of surgical resection. Residual disease was defined by the presence of microscopic (R1) or macroscopic (R2) residual tumor at the margin.

A safety lead-in period was conducted to evaluate surgical feasibility. Cohort B enrollment was reopened after the safety lead-in period, and Cohort A enrollment was reopened after the enrollment of 8 patients with PD-L1 tumor expression of 50% or higher in Cohort B. All subsequent patients with PD-L1 tumor expression of 50% or higher were enrolled in Cohort A.

### End points and Assessments

The primary end points of SKYSCRAPER-05 were the safety of perioperative tiragolumab plus atezolizumab, based on the incidence and severity of adverse events (AEs), surgical feasibility and surgical outcomes, and the efficacy of perioperative treatment as assessed by the proportion of patients achieving a major pathologic response (MPR) by local pathology. MPR was defined as 10% or less residual viable tumor at the time of surgical resection in the primary tumor, as assessed by a local pathology laboratory. Surgical feasibility was evaluated based on the proportion of patients who underwent complete resection (R0), the types of surgeries performed, and delays or cancellations because of AEs or disease progression.

Secondary end points included pathologic complete response (pCR) rate by local pathology, defined as the absence of any viable TC in both the primary tumor and all sampled lymph nodes at the time of surgical resection, and investigator-assessed event-free survival (EFS). Patient-reported outcomes (PROs) were exploratory end points.

All patients completed scheduled tumor assessments of the chest and abdomen by computed tomography (CT) and positron emission tomography scans at screening and by CT scan after cycles 2 and 4 of neoadjuvant therapy to determine treatment response. The investigator reviewed the scan results after cycle 2 to determine whether the patient was eligible to receive cycle 3 of neoadjuvant treatment or if the patient should proceed with surgery (for those with disease progression but still amenable to curative surgery) or other standard of care treatment (for those with disease progression not amenable to curative surgery). Following surgery, CT scans were performed every 4 months for the first year after the procedure and every 6 months in the second year until recurrence.

The severity of AEs was assessed according to the National Cancer Institute Common Terminology Criteria for AEs v5.0. PROs of physical functioning and role functioning were assessed in Cohort B only using the European Organisation for the Research and Treatment of Cancer (EORTC) core Quality of Life Questionnaire (QLQ-C30)^17^^,^^18^ and were assessed at cycles 1–5, every other cycle thereafter, and at treatment discontinuation.

### Statistical Analysis

Assuming an observed MPR rate of 61%, a minimum of 41 patients needed to be enrolled in Cohort B for the point estimate of the MPR rate to have a two-sided 95% confidence interval (CI) lower bound that exceeded 46%, a rate that may warrant further investigation of the combination therapy in this setting. The same number of patients was originally planned for enrollment in Cohort A.

All safety and efficacy analyses were descriptive and conducted in patients who received at least one dose of perioperative study treatment. MPR and pCR rates were reported with two-sided 95% CIs calculated using the Wilson method. Patients who did not undergo surgery were considered nonresponders to MPR. EFS curves and rates were evaluated using the Kaplan‒Meier method for each treatment cohort. Descriptive statistics were used to summarize baseline characteristics, surgical outcomes, and AEs.

## Results

### Patient Disposition

Between April 23, 2021, and September 14, 2023, 50 patients were enrolled at 20 sites (Cohort A, n = 8; Cohort B, n = 42). Enrollment in Cohort A was discontinued early following a shift in the treatment landscape for resectable NSCLC, as data became available from phase 3 trials of perioperative regimens combining chemotherapy with immunotherapy that showed improved efficacy outcomes compared with chemotherapy alone.^1^, ^2^, ^3^, ^4^ Therefore, this study will primarily focus on the results and discussion of Cohort B.

**Figure 1:**
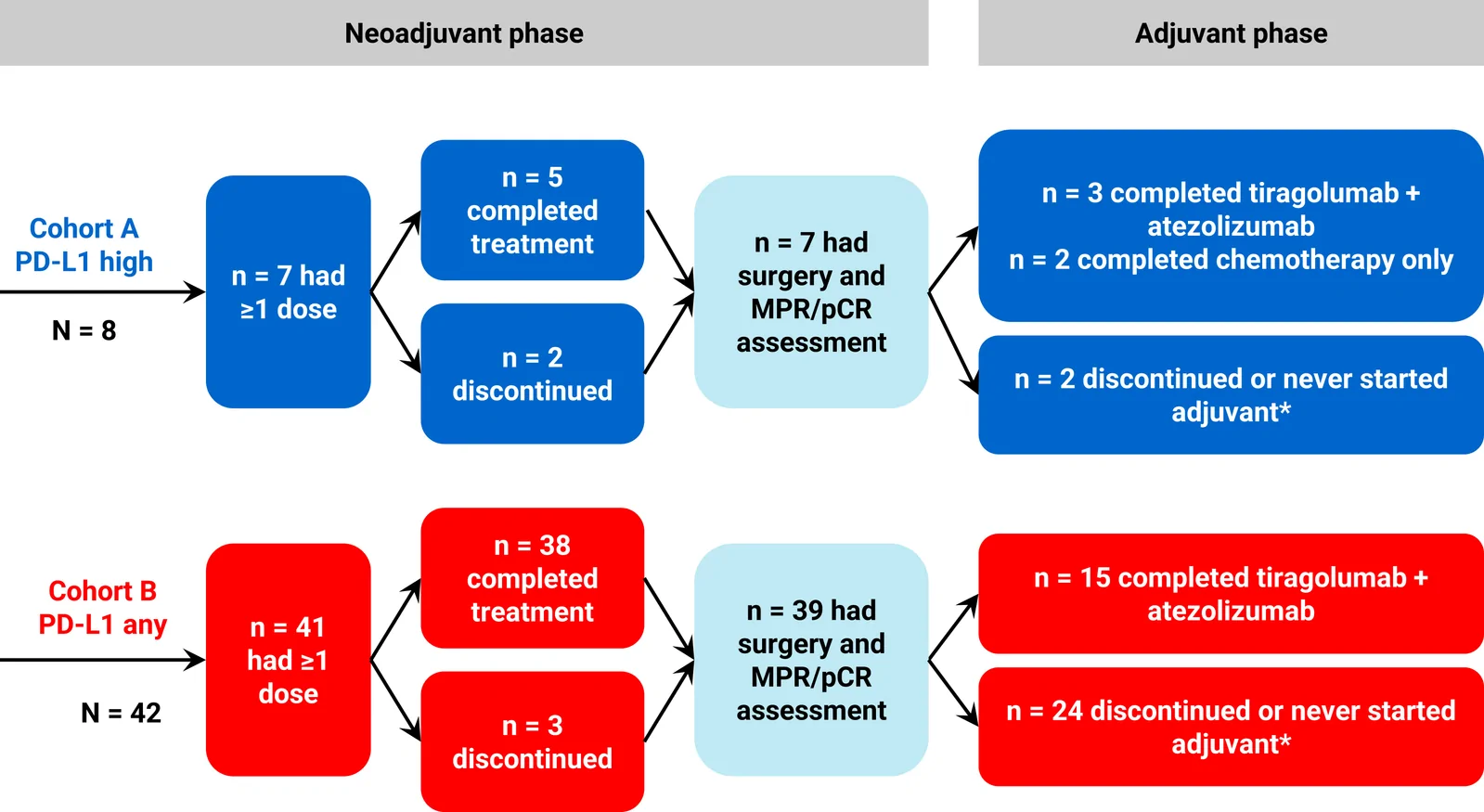
Summary of patient disposition. ∗Includes one patient who discontinued perioperative treatment. MPR, major pathologic response; pCR, pathologic complete response; PD-L1, programmed death-ligand 1.

Baseline demographic and patient characteristics are summarized in Table 1. Most patients were male (70%), White (66%), and aged 65 years or older (66%). In Cohort B, 57%, 24%, and 19% of patients had tumors with PD-L1 TC expression levels of less than 1%, 1% to 49%, and 50% or higher, respectively. The efficacy- and safety-evaluable populations comprised 48 patients who received at least one dose of perioperative study treatment (Cohort A, n = 7; Cohort B, n = 41; Fig. 1). In Cohort A, 71% of patients completed the neoadjuvant treatment phase, and 43% completed both neoadjuvant and adjuvant treatment phases, whereas in Cohort B, 93% completed the neoadjuvant treatment phase, and 37% completed the adjuvant treatment phase. One patient in Cohort A did not complete neoadjuvant tiragolumab plus atezolizumab but continued in the study on the advice of the investigator because they had a small increase in target lesion size when assessed at the end of Cycle 2; the patient underwent surgery and received adjuvant tiragolumab plus atezolizumab. The main reasons for treatment discontinuation were physician decision (29%) in Cohort A and AEs (32%; not exclusively study drug-related) in Cohort B.

**Table 1:** Demographic and Baseline Characteristics

n (%)	Cohort A PD-L1 high (n = 8)	Cohort B PD-L1 any (n = 42)	All patients (N = 50)
Age group (years)			
<65	2 (25)	15 (36)	17 (34)
≥65	6 (75)	27 (64)	33 (66)
Sex			
Male	6 (75)	29 (69)	35 (70)
Female	2 (25)	13 (31)	15 (30)
Race			
Asian	0	16 (38)	16 (32)
White	8 (100)	25 (60)	33 (66)
Unknown	0	1 (2)	1 (2)
Histology			
Squamous	2 (25)	25 (60)	27 (54)
Non-squamous	6 (75)	17 (40)	23 (46)
Stage			
II	3 (38)	17 (40)	20 (40)
IIIA N2^+^	3 (38)	9 (21)	12 (24)
IIIA N2^–^	1 (13)	14 (33)	15 (30)
IIIB	1 (13)	2 (5)	3 (6)
PD-L1 expression			
TC <1%	0	24 (57)	24 (48)
TC 1%–49%	0	10 (24)	10 (20)
TC ≥50%	8 (100)	8 (19)	16 (32)

PD-L1 status was determined using the VENTANA PD-L1 (SP263) immunohistochemistry assay analyzed by a central laboratory.

PD-L1, programmed death-ligand 1; TC, tumor cell.

### Safety

Exposure to study treatment is summarized in Supplementary Table 1. Exposure to adjuvant treatment was used to delineate the neoadjuvant and adjuvant phases in the safety analysis. At the time of clinical cutoff for safety (March 5, 2025; median safety follow-up of 15.1 months), the proportion of patients who had experienced at least one AE in Cohort A and B was 100% and 98%, respectively, during neoadjuvant treatment, and 100% and 91%, respectively, during adjuvant treatment (Table 2). Most reported events during both treatment phases in the cohorts were Grade 1 to 2. Specifically, Grade 1 to 2 AEs occurred in 57% (n = 4) of patients in Cohort A and 54% (n = 22) in Cohort B during neoadjuvant treatment, and in 100% (n = 5) and 58% (n = 19) of patients, respectively, during adjuvant treatment. The most common AEs were alanine aminotransferase increased, aspartate aminotransferase increased, and anemia (all 43%) in Cohort A, and rash (32%), diarrhea, and anemia (both 24%) in Cohort B during the neoadjuvant phase, and pruritus (60%) in Cohort A, and rash (18%) in Cohort B during the adjuvant phase (Supplementary Table 2).

**Table 2:** Overall Safety Summary During the Neoadjuvant and Adjuvant Treatment Periods

Neoadjuvant treatment period, n (%)	Cohort A PD-L1 high (n = 7)	Cohort B PD-L1 any (n = 41)	All patients (N = 48)
Patients with at least one AE	7 (100)	40 (98)	47 (98)
Total number of AEs	47	369	416
Patients with at least one of the following:			
Treatment-related AE			
Any treatment	7 (100)	38 (93)	45 (94)
Tiragolumab	7 (100)	33 (81)	40 (83)
Atezolizumab	7 (100)	32 (78)	39 (81)
Chemotherapy	0	34 (83)	34 (71)
Grade 3/4 AE	3 (43)	16 (39)	19 (40)
Treatment-related Grade 3/4 AE	2 (29)	13 (32)	15 (31)
Grade 5 AE	0	2 (5)^a^	2 (4)
Treatment-related Grade 5 AE	0	1 (2)	1 (2)
Serious AE	2 (29)	7 (17)	9 (19)
Treatment-related serious AE	1 (14)	1 (2)	2 (4)
AE leading to withdrawal from treatment			
Any treatment	0	4 (10)	4 (8)
Tiragolumab	0	3 (7)	3 (6)
Atezolizumab	0	3 (7)	3 (6)
Chemotherapy	0	2 (5)	2 (4)
AE leading to dose modification or interruption			
Any treatment	1 (14)	14 (34)	15 (31)
Tiragolumab	1 (14)	7 (17)	8 (17)
Atezolizumab	1 (14)	8 (20)	9 (19)
Chemotherapy	0	11 (27)	11 (23)

Adjuvant treatment period, n (%)	Cohort A PD-L1 high (n = 5)	Cohort B PD-L1 any (n = 33)	All patients (N = 38)
Patients with at least one AE	5 (100)	30 (91)	35 (92)
Total number of AEs	46	185	231
Patients with at least one of the following:			
Treatment-related AE			
Any treatment	5 (100)	24 (73)	29 (76)
Tiragolumab	4 (80)	22 (67)	26 (68)
Atezolizumab	4 (80)	23 (70)	27 (71)
Chemotherapy	2 (40)	7 (21)	9 (24)
Grade 3/4 AE	0	9 (27)	9 (24)
Treatment-related Grade 3/4 AE	0	7 (21)	7 (18)
Grade 5 AE	0	2 (6)^b^	2 (5)
Treatment-related Grade 5 AE	0	1 (3)	1 (3)
Serious AE	0	7 (21)	7 (18)
Treatment-related serious AE	0	4 (12)	4 (11)
AE leading to withdrawal from treatment			
Any treatment	0	11 (33)	11 (29)
Tiragolumab	0	11 (33)	11 (29)
Atezolizumab	0	11 (33)	11 (29)
Chemotherapy	0	0	0
AE leading to any dose modification or interruption			
Any treatment	2 (40)	11 (33)	13 (34)
Tiragolumab	2 (40)	11 (33)	13 (34)
Atezolizumab	2 (40)	11 (33)	13 (34)
Chemotherapy	0	0	0

AE, adverse event; PD-L1, programmed death-ligand 1.

a Infectious pleural effusion related to chemotherapy.

b Acute pyelonephritis unrelated to treatment and unexplained death.

AEs assessed as treatment-related by investigators occurred in 100% and 93% of patients in Cohort A and B, respectively, during the neoadjuvant phase, and in 100% and 73% of patients in Cohort A and B, respectively, during the adjuvant phase. Grade 3/4 treatment-related AEs occurred in 29% and 32% of patients in Cohort A and B, respectively, during the neoadjuvant phase, and in 0% and 21% of patients during the adjuvant phase (Table 2). Serious AEs were reported in 29% of patients in Cohort A and 17% in Cohort B during the neoadjuvant phase; the corresponding proportions during the adjuvant phase were 0% (Cohort A) and 21% (Cohort B).

Grade 5 AEs occurred in 2 patients (4%) during neoadjuvant treatment (infectious pleural effusion related to chemotherapy) and in 2 patients (5%) during adjuvant treatment (acute pyelonephritis unrelated to treatment and an unexplained death), all in Cohort B. The unexplained death occurred in a 74-year-old female who presented to the emergency room with abdominal pain and vomiting and soon experienced cardiac arrest; a potential relationship with tiragolumab plus atezolizumab treatment could not be ruled out by the investigator. An additional Grade 5 AE of respiratory distress occurred in 1 patient in Cohort B after surgery and was considered surgery-related (Table 2). The proportion of patients who experienced an AE leading to withdrawal from any study treatment in Cohorts A and B was 0% and 10%, respectively, during neoadjuvant treatment, and 0% and 33%, respectively, during adjuvant treatment.

AEs of special interest (AESIs) were experienced by 71% and 68% of patients in Cohorts A and B, respectively, during the neoadjuvant phase, and by 40% and 60% of patients, respectively, during the adjuvant phase (Supplementary Table 3). The proportion of patients with Grade 3/4 AESIs in Cohorts A and B was 29% and 5%, respectively, in the neoadjuvant phase, and 0% and 15%, respectively, in the adjuvant phase. No Grade 5 AESIs were reported. The most common AESIs were immune-mediated rash in both Cohort A (57%) and Cohort B (42%) during the neoadjuvant phase, and immune-mediated hypothyroidism in Cohort A (40%) and immune-mediated rash in Cohort B (27%) during the adjuvant phase. AESIs requiring systemic corticosteroids were experienced by 14% and 29% of patients in Cohort A and B, respectively, during the neoadjuvant phase, and by 20% and 21% of patients in Cohort A and B, respectively, during the adjuvant phase. The proportion of patients with AESIs requiring immunosuppressants other than corticosteroids in Cohort A and B was 14% and 0%, respectively, during the neoadjuvant phase, and 0% and 3%, respectively, during the adjuvant phase.

### Surgical Outcomes

At the time of clinical cutoff (March 5, 2025; median safety follow-up, 15.1 months), all patients in Cohort A (7/7) and 95% of patients in Cohort B (37/39) had undergone surgical resection (Table 3). All patients in Cohort A and 90% of patients in Cohort B had undergone a lobectomy or bilobectomy, with 86% of patients in Cohort A and 95% of patients in Cohort B having an R0 resection. The proportions of patients undergoing other types of surgery and with R1 and R2 resection margin statuses are summarized in Table 3. Surgery was not performed in 5% of patients in Cohort B because of progressive disease, and investigators delayed surgery in 10% of patients in Cohort B because of AEs (thrombocytopenia, n = 1; dyspnea, n = 1) and scheduling issues (n = 2; Table 3). No surgery delays or cancellations were reported in Cohort A.

**Table 3:** Surgical Safety

	Cohort A PD-L1 high (n = 7)	Cohort B PD-L1 any (n = 41)	All patients (N = 48)
Patients in whom surgery was not performed	0	2 (5)	2 (4)
Reason for surgery not performed			
Progressive disease	0	2 (5)	2 (4)
Patients in whom surgery was delayed	0	4 (10)	4 (8)
AE	0	2 (5)^a^	2 (4)^a^
Other	0	2 (5)^b^	2 (4)^b^
Patients undergoing surgery	*n= 7*	*n= 39*	*n= 46*
Type of resection			
Pneumonectomy	0	2 (5)^c^	2 (4)^c^
Bilobectomy	0	3 (8)^c^	3 (7)^c^
Lobectomy	7 (100)	32 (82)^c^	39 (85)^c^
Segmentectomy	0	1 (3)^c^	1 (2)^c^
Other	0	1 (3)^c^	1 (2)^c^
Residual tumor classification			
R0	6 (86)	37 (95)^c^	43 (93)^c^
R1	1 (14)	2 (5)^c^	3 (7)^c^
R2	0	0^c^	0^c^
Patients with increased perihilar/lobar adhesions^d^	3 (43)	22 (56)^c^	25 (54)^c^
Grade 1	2 (29)	5 (13)^c^	7 (15)^c^
Grade 2	1 (14)	9 (23)^c^	10 (22)^c^
Grade 3	0	8 (21)^c^	8 (17)^c^
Length of hospitalization for surgery, days			
Mean	8.3	8.5	8.5
Median	3.0	6.0	6.0
Min−Max	1.0−36.0	2.0−69.0	1.0−69.0
Patients with intraoperative complications			
Pulmonary artery injury	0	3 (7)^c^	3 (6)^c^
Patients with post-operative complications	2 (29)	7 (18)	9 (20)
Pneumonia	1 (14)	2 (5)	3 (7)
Bronchopleural fistula	0	1 (3)	1 (3)
Initial ventilator support >48 hours	0	1 (3)	1 (3)
Reintubation	0	1 (3)	1 (3)
Tracheostomy	0	1 (3)	1 (3)
Added instrumentation (e.g., swan, chest tube, ECMO)	1 (14)	4 (10)	5 (11)
Patients going home with chest tube	0	2 (5)	2 (4)
Chest tube duration, days			
Median	2.0	3.0	3.0
Min−Max	1.0−25	1.0−68	1−68

AE, adverse event; ECMO, extracorporeal membrane oxygenation; Max, maximum; Min, minimum; PD-L1, programmed death-ligand 1.

a Thrombocytopenia and dyspnea.

b Surgical delay because of scheduling issues.

c Percentages were calculated using the total number of patients who underwent surgery (n = 39).

d Grade 1 was defined as mild fibrosis (having no significant impact on the conduct of surgical resection), grade 2 was defined as moderate fibrosis (requiring increased effort and dissection during resection but otherwise not severely impacting the conduct of the surgery), grade 3 was defined as severe fibrosis (significantly impacting the conduct of the operation by increasing the duration or blood loss during the surgery or requiring conversion from minimally invasive to open surgery).

Surgery-related AEs (i.e., occurring within 30 days after surgery) were reported in 57% of patients in Cohort A and 36% in Cohort B and were Grade 3/4 in severity in 14% and 3% of patients, respectively. Intraoperative complications were reported in patients in Cohort B only (7%) and involved the pulmonary artery (Table 3). Increased perihilar/lobar adhesions occurred in 43% of patients in Cohort A and 54% of patients in Cohort B, with Grade 3 adhesions in 0% and 20% of patients, respectively.

### Efficacy

**Figure 2:**
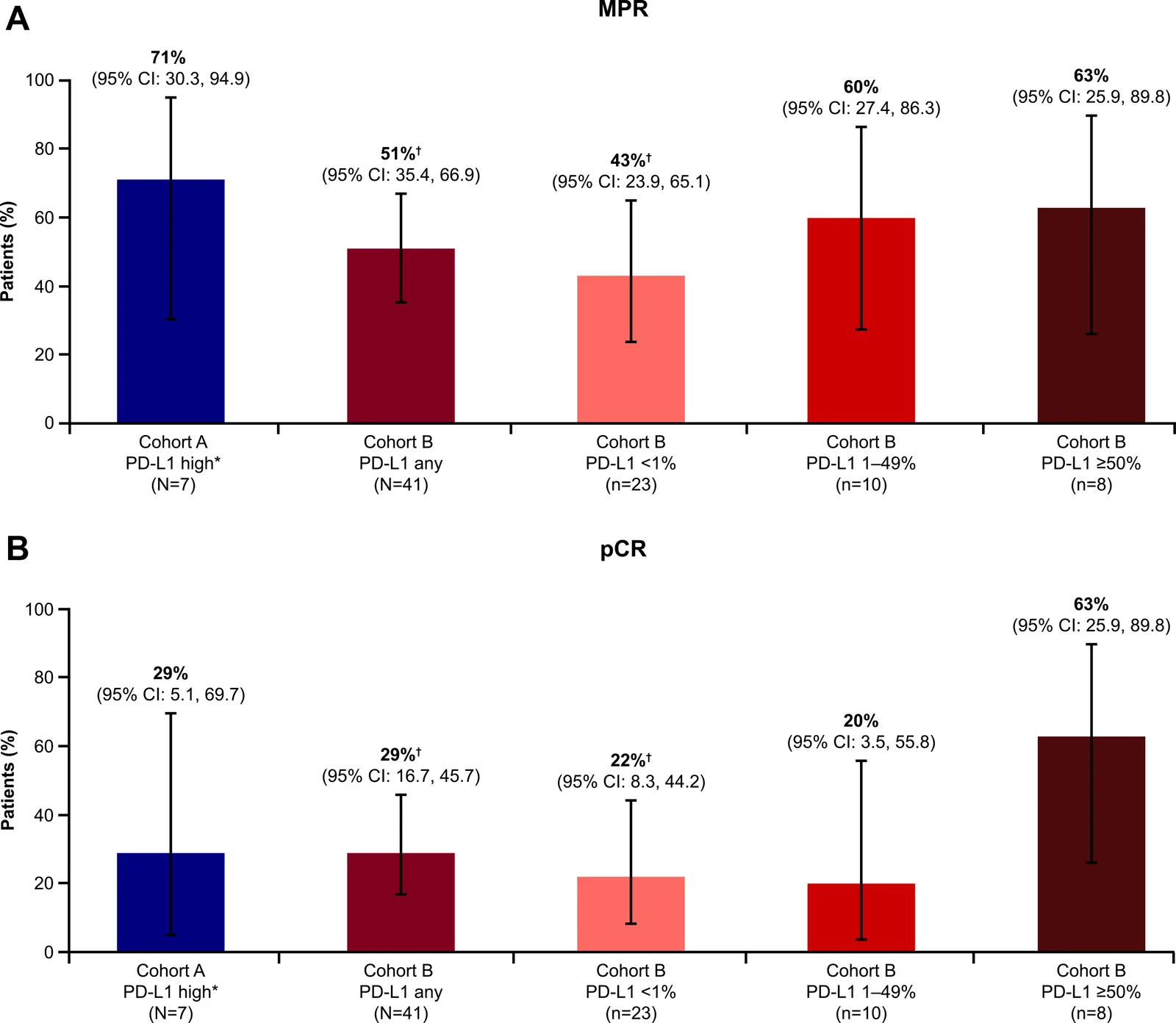
MPR (*A*) and pCR (*B*) rates overall and by PD-L1 TC subgroup in Cohort B. ∗PD-L1 high defined as TC ≥ 50%. ^†^Two patients did not undergo surgery and were counted as non-MPR and non-pCR. CI, confidence interval; MPR, major pathologic response; pCR, pathologic complete response; PD-L1, programmed death-ligand 1; TC, tumor cell.

At a median follow-up of 9.5 months (clinical cutoff: February 14, 2024), in Cohort A (n = 7), the MPR and pCR rates were 5/7 (71%; 95% CI: 30‒95) and 2/7 (29%; 95% CI: 5‒70), respectively. In Cohort B (n = 41), the MPR and pCR rates were 51% (95% CI: 35‒67) and 29% (95% CI: 17‒46), respectively. MPR and pCR were observed across all PD-L1 TC subgroups examined and were highest in the PD-L1 TC 50% or higher subgroup (Fig. 2).

In Cohort A, the overall response rate and CR rate were 4/7 (57%, 95% CI: 20‒88) and 1/7 (14%, 95% CI: 1‒58), respectively (Supplementary Table 4). In Cohort B, the overall response rate and CR rate were 27/41 (66%, 95% CI: 49‒79) and 1/41 (2%, 95% CI: 0‒14), respectively (Supplementary Table 4).

**Figure 3:**
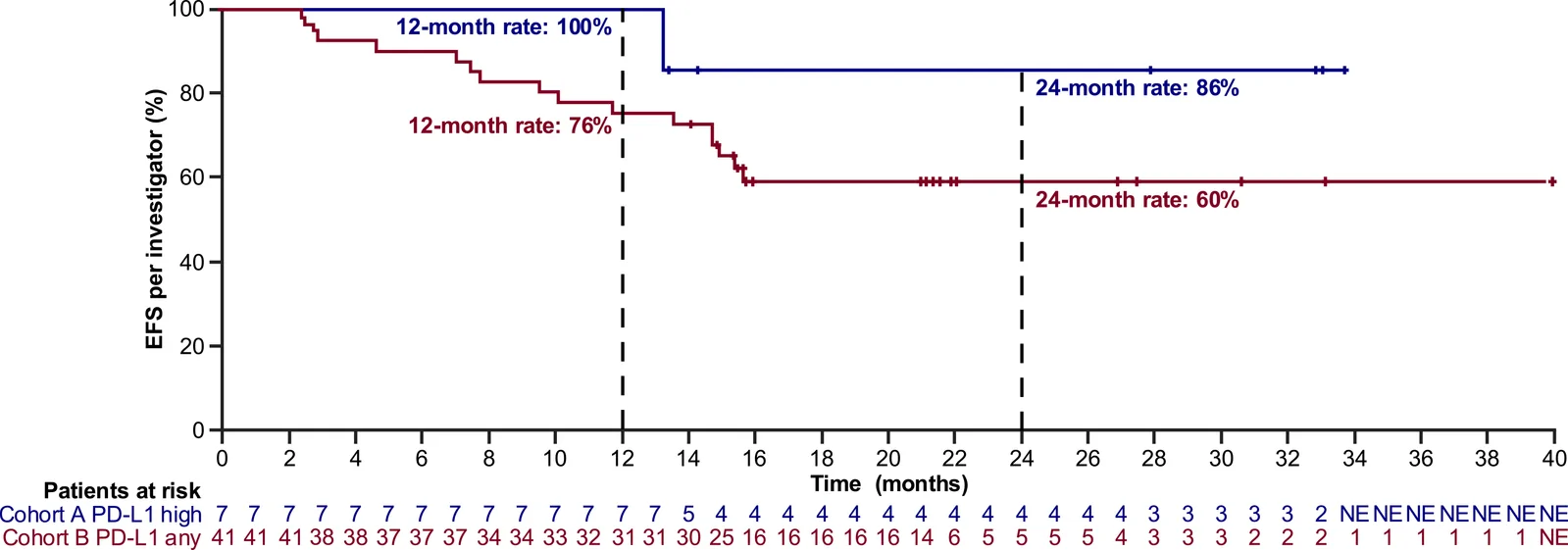
Kaplan‒Meier curves of EFS in Cohorts *A* and *B*. EFS, event-free survival; NE, not estimable; PD-L1, programmed death-ligand 1.

As of March 5, 2025 (median follow-up: all patients, 22.2 months; Cohort A, 33.3 months; Cohort B, 20.1 months), the median EFS was not estimable in either cohort (Fig. 3). The estimated landmark EFS rates in Cohort A and B were 100% and 76% at 12 months, and 86% and 60%, at 24 months, respectively (Fig. 3).

### PROs

In Cohort B, QLQ-C30 scores for mean physical functioning and role functioning (scale 0–100, where higher scores indicate better functioning) were greater than 85 at baseline (n = 40; Supplementary Fig. 1 and 2). Mean scores for physical and role functioning decreased to greater than 75 after neoadjuvant treatment (n = 40) and decreased to approximately 70 and 55 for physical and role functioning, respectively, post-surgery (n = 33). By cycle 9 of adjuvant treatment, both scales were greater than 80 in Cohort B (n = 28), returning to baseline levels and consistent with population norms.^18^

## Discussion

SKYSCRAPER-05 was designed to establish proof-of-concept of clinical safety, surgical feasibility, and efficacy for perioperative treatment with either tiragolumab plus atezolizumab (Cohort A) or tiragolumab plus atezolizumab plus platinum-based chemotherapy (Cohort B) in patients with locally advanced, resectable NSCLC. Enrollment into Cohort A was terminated after only 8 patients were enrolled because of external data and shifting practice patterns following the approval of perioperative checkpoint inhibitors plus chemotherapy. Consequently, the primary focus of the study was Cohort B.

The safety profile of perioperative tiragolumab plus atezolizumab with or without platinum-based chemotherapy was consistent with the known profiles of these agents, and no new or unexpected safety findings were observed. Notably, the addition of a TIGIT inhibitor did not lead to an escalation of high-grade toxicities that might otherwise jeopardize the timing of surgery. This is reported by the fact that Grade 3/4 treatment-related AEs in SKYSCRAPER-05 remained within the 33%–45% range observed in landmark phase 3 trials such as CheckMate 816 and KEYNOTE-671.^1^^,^^4^

Surgical feasibility was a key finding in the neoadjuvant setting, with 96% of patients undergoing surgical resection–a rate that compares favorably with the 77% to 83% resection rates reported in the AEGEAN, KEYNOTE-671, and CheckMate 816 studies.^1^^,^^2^^,^^4^ These findings suggest that the TIGIT/PD-L1 combination does not result in an excess of immune-mediated toxicities or disease progression that would preclude patients from definitive surgery. Furthermore, the 93% R0 resection rate and the low incidence of intraoperative complications, consistent with the 89% to 95% R0 rates observed in recent phase 3 perioperative studies, confirm that surgical quality may be maintained with this regimen.^1^, ^2^, ^3^, ^4^ The manageable recovery profile is further supported by data from functional scales, as measured by the QLQ-C30, which showed that average physical functioning and role functioning returned to population norm values after surgery among patients who remained on treatment, with no detrimental impact on quality of life.

In Cohort B, the MPR and pCR rates were 51% and 29%, respectively. These results are comparable to those reported in phase 3 studies investigating other perioperative immunotherapy plus chemotherapy regimens in patients with resectable NSCLC, where MPR rates ranged from 30% to 57%, and pCR rates ranged from 17% to 37%.^1^, ^2^, ^3^, ^4^^,^^15^^,^^19^^,^^20^ Notably, in our study and across the aforementioned trials, clinical benefit was observed across all PD-L1 expression subgroups, including those with low PD-L1 expression, although the greatest benefits were observed in PD-L1-high subpopulations. Because of the small number of patients in each subgroup, it is difficult to draw meaningful conclusions regarding a potential correlation between radiographic and pathologic response.

These collective findings from SKYSCRAPER-05 should be interpreted with caution because of the small number of patients and the inherent limitations of cross-study comparisons. Despite these caveats, the data from Cohort B align with global trends and provide evidence for the efficacy of this combination in the perioperative setting.

More studies are needed to identify neoadjuvant therapy predictive factors for MPR and pCR in resectable NSCLC and to determine the optimal patient selection for adjuvant therapy. In addition, more data are required to identify patients who can benefit from immunotherapy alone in the neoadjuvant setting, potentially sparing them from the toxicities of chemotherapy. Furthermore, long-term follow-up data for overall survival are of utmost importance in trials of perioperative targeted therapies or immunotherapy-based regimens to determine the durability of the treatment effect and the impact of late toxicity on patient outcomes.

### Limitations

As an exploratory phase 2 study, SKYSCRAPER-05 was designed to explore safety and surgical feasibility and to identify signals of treatment efficacy without a control arm. In addition, the early closure of Cohort A resulted in very small patient numbers and limited the interpretation of the data for the tiragolumab plus atezolizumab arm. Interpretation of the findings was further impacted by the immaturity of the EFS data at the clinical cutoff date, which prevented a definitive assessment of survival outcomes.

## Conclusions

Results from SKYSCRAPER-05 confirmed the surgical feasibility of neoadjuvant tiragolumab plus atezolizumab, with or without platinum-based chemotherapy, for patients with locally advanced, resectable NSCLC. Tiragolumab plus atezolizumab reported an acceptable safety profile consistent with expectations for this combination regimen in a resectable disease setting. MPR and pCR rates were observed across PD-L1 TC subgroups with perioperative tiragolumab plus atezolizumab that were similar to those of other perioperative immunotherapy plus chemotherapy regimens.

## CRediT Authorship Contribution Statement

**Catherine A. Shu:** Investigation, Writing – original draft, Writing – review & editing.

**Anthony W. Kim:** Investigation, Writing – original draft, Writing – review & editing.

**Min Hee Hong:** Investigation, Writing – original draft, Writing – review & editing.

**Alex Martinez Marti:** Investigation, Writing – original draft, Writing – review & editing.

**Martin Früh**: Investigation, Writing – original draft, Writing – review & editing.

**Andrea Ferris:** Investigation, Writing – original draft, Writing – review & editing.

**Harvey Pass**: Investigation, Writing – original draft, Writing – review & editing.

**Bryan A. Chan:** Investigation, Writing – original draft, Writing – review & editing.

**Saiama N. Waqar:** Investigation, Writing – original draft, Writing – review & editing.

**Coen A. Bernaards**: Formal analysis, Validation, Writing – original draft, Writing – review & editing.

**Namrata S. Patil:** Formal analysis, Validation, Writing – original draft, Writing – review & editing.

**Jessie J. Hsu:** Formal analysis, validation, writing – original draft, writing – review & editing.

**Cristal Parsons:** Formal analysis, Validation, Writing – original draft, Writing – review & editing.

**Sarah Troutman:** Formal analysis, Validation, Writing – original Draft, Writing – review & editing.

**Christina Matheny:** Formal analysis, Validation, Writing – original draft, Writing – review & editing.

**Pao-Chen Li**: Formal analysis, Validation, Writing – original draft, Writing – review & editing.

**Barbara Gitlitz:** Formal analysis, Validation, Writing – original draft, Writing – review & editing.

**Enriqueta Felip**: Investigation, Writing – original draft, Writing – review & editing.

## Data sharing statement

For up-to-date details on Roche's global policy on the sharing of clinical information and how to request access to related clinical study documents, see here: https://go.roche.com/data_sharing. Anonymized records for individual patients across more than one data source external to Roche cannot and should not be linked because of a potential increase in the risk of patient re-identification.

## Disclosure

Dr. Catherine A. Shu has received travel support from Genentech; and participated in a data safety monitoring board/advisory board for AstraZeneca, Johnson & Johnson, EMD Serono, and Gilead in the past 36 months. Dr. Anthony W. Kim has received honoraria from Medtronic in the past 36 months. Mr. Min Hee Hong has received grants or contracts from AstraZeneca, Novartis, MSD, and Yuhan; honoraria from AstraZeneca, Amgen, Bristol Myers Squibb, MSD, Ono, Takeda, Roche, Pfizer, and Yuha; has held stocks/stock options at GI Cell and GI Biom; and reports other financial or non-financial interests with AbbVie, IMPACT, Ignyte, Loxo Oncology, Novartis, Merck, ORIC, AstraZeneca, Bristol Myers Squibb, MSD, and Pfizer in the past 36 months. Mr. Alex Martinez-Marti has received consulting fees from Amgen, AstraZeneca, Boehringer Ingelheim, Bristol Myers Squibb, F. Hoffmann-La Roche AG, Fuchigec, Lilly, MedImmune, MSD, MSD Oncology, and Pfizer; and honoraria and travel support from Amgen, AstraZeneca, Boehringer Ingelheim, Bristol Myers Squibb, F. Hoffmann-La Roche AG, Lilly, MedImmune, MSD, MSD Oncology, and Pfizer in the past 36 months. Mr. Martin Früh has received fees for participation on advisory boards of BMS, MSD, AstraZeneca, Boehringer Ingelheim, Roche, Takeda, Pfizer, Janssen, Daiichi Sankyo, and Pharmamar; and has had a leadership role (head of the Scientific Committee) at the Swiss Cancer Institute in the past 36 months. Mrs. Andrea E. Ferris has served as president/CEO of the LUNGevity Foundation in the past 36 months. Dr. Harvey Pass has received grants or contracts from Roche/Genentech in the past 36 months. Mr. Bryan Anthony Chan has received consulting fees from AstraZeneca, Daiichi Sankyo, MSD, and Roche; honoraria from AstraZeneca, MSD, and Roche; and travel support from MSD and Roche in the past 36 months. Dr. Saiama N. Waqar has received grants or contracts from SWOG Clinical trial partnership, Nuvalent, AstraZeneca, AbbVie, Ariad Pharmaceuticals, Genentech, Immunomedics Inc., Roche, Astellas Pharma Inc, Daiichi Sankyo, Verastem Inc, Janssen Research & Development LLC, Elevation Oncology, Loxo Oncology, and Takeda Pharmaceuticals; honoraria from OncLive, DAVA Oncology, ASCO, Karmanos Cancer Center, and NCC; consulting fees from Gilead and AstraZeneca; travel support from SWOG, IASLC, NYLCF; received fees for participation on advisory boards of AstraZeneca, Pfizer, Amgen, Daiichi Sankyo, Boehringer Ingelheim, Gilead, Janssen, Adcendo, and MSD; and has served as the Chair of the Data Safety Monitoring Board for a specific HCRN study in the past 36 months. Mr. Coen A. Bernaards, Dr. Namrata S. Patil, Ms. Jessie J. Hsu, Ms. Cristal Parsons, Ms. Sarah Troutman, Dr. Christina Matheny, Dr. Pao-Chen Li and Dr. Barbara Gitlitz are employees of Genentech and have held stocks/stock options at Genentech/Roche in the past 36 months. Dr. Enriqueta Felip has received consulting fees from AbbVie, Amgen, AstraZeneca, Boehringer Ingelheim, Bristol Myers Squibb, Daiichi Sankyo, Genentech/Roche, GlaxoSmithKline, Iteos Therapeutics, Janssen/Johnson & Johnson, MSD, Pierre Fabre, Pfizer, Pharmamar, Seagen, Regeneron, Tubulis, Moderna, and Ellipses Pharma; honoraria from Amgen, Daiichi Sankyo, Eli Lilly, Roche/Genentech, Gilead, Janssen/Johnson & Johnson, Medical Trends, Medscape, MSD, Peervoice, and Pfizer; travel support from AstraZeneca, Johnson & Johnson, Roche, and Amgen; and has been an independent board member of Grifols in the past 36 months. Pao-Chen Li declares no conflict.
