# Supplementary material for: SKYSCRAPER-05: A Phase 2 Study of Perioperative Tiragolumab Plus Atezolizumab, With or Without Platinum-Based Chemotherapy, in Patients With Previously Untreated, Locally Advanced, Resectable, Stage II‒IIIB NSCLC
Source: JTO Clin Res Rep. 2026 Jul 10;7(10):101047. doi: 10.1016/j.jtocrr.2026.101047 (PMC13577865; doi:10.1016/j.jtocrr.2026.101047)
Supplement: Supplementary file 1 [file mmc1.docx]

# **SUPPLEMENTARY MATERIALS**

**Supplementary Table 1.** Treatment exposure.

|  | Cohort A PD-L1 high  (n = 7) | Cohort B PD-L1 any  (n = 41) | All patients  (N = 48) |
| --- | --- | --- | --- |
| Treatment duration, months |  |  |  |
| Mean (SD) | 7.70 (7.75) | 10.69 (5.52) | 10.25 (5.89) |
| Median | 2.46 | 13.01 | 12.67 |
| Min–Max | 0.8–16.5 | 0.0–18.2 | 0.0–18.2 |
| Total cumulative dose, mg |  |  |  |
| Mean (SD) | 12342.9 (10948.2) | 16068.3 (8147.1) | 15525.0 (8576.5) |
| Median | 4800.0 | 18000.0 | 18000.0 |
| Min–Max | 2400–24000 | 1200–24000 | 1200–24000 |
| Treatment duration, % |  |  |  |
| 0 to ≤3 months | 4 (57) | 8 (20) | 12 (25) |
| >3 to ≤6 months | 0 | 1 (2) | 1 (2) |
| >6 to ≤12 months | 0 | 10 (24) | 10 (21) |
| >12 months | 3 (43) | 22 (54) | 25 (52) |
| Dose intensity, % |  |  |  |
| Mean (SD) | 90.79 (8.40) | 83.16 (10.99) | 84.27 (10.92) |
| Median | 88.42 | 85.07 | 85.13 |
| Min–Max | 80.6–102.4 | 64.4–102.4 | 64.4–102.4 |
| Doses received (pre-surgery), n |  |  |  |
| Mean (SD) | 3.4 (1.0) | 3.9 (0.6) | 3.8 (0.7) |
| Median | 4.0 | 4.0 | 4.0 |
| Min–Max | 2–4 | 1–4 | 1–4 |
| Doses received (post-surgery), n | *n = 3* | *n = 33* | *n = 36* |
| Mean (SD) | 16.0 (0.0) | 11.8 (5.0) | 12.2 (5.0) |
| Median | 16.0 | 15.0 | 15.5 |
| Min–Max | 16–16 | 1–16 | 1–16 |

Treatment duration was calculated as the date of the last dose of study medication minus the date of the first dose plus one day.

Dose intensity was calculated as the sum of (actual dose at each cycle/planned dose at cycle 1, day 1) divided by [(last dosing date – first dosing date/21)+1] multiplied by 100.

Max, maximum; Min, minimum; PD-L1, programmed death-ligand 1; SD, standard deviation.

**Supplementary Table 2.** Most common AEs (incidence ≥10%) during the neoadjuvant and adjuvant treatment periods by MedDRA system organ class and preferred term.

| **Neoadjuvant treatment period, n (%)** | Cohort A  PD-L1 high  (n = 7) | Cohort B  PD-L1 any  (n = 41) | All patients  (N = 48) |
| --- | --- | --- | --- |
| Gastrointestinal disorders |  |  |  |
| Diarrhea | 1 (14) | 10 (24) | 11 (23) |
| Constipation | 2 (29) | 7 (17) | 9 (19) |
| Nausea | 0 | 8 (20) | 8 (17) |
| Vomiting | 0 | 5 (12) | 5 (10) |
| Skin and subcutaneous tissue disorders |  |  |  |
| Rash | 2 (29) | 13 (32) | 15 (31) |
| Pruritus | 2 (29) | 8 (20) | 10 (21) |
| Rash maculo-papular | 1 (14) | 1 (2) | 2 (4) |
| Skin toxicity | 1 (14) | 0 | 1 (2) |
| Investigations |  |  |  |
| Alanine aminotransferase increased | 3 (43) | 8 (20) | 11 (23) |
| Aspartate aminotransferase increased | 3 (43) | 8 (20) | 11 (23) |
| Gamma glutamyl-transferase increased | 1 (14) | 0 | 1 (2) |
| SARS-CoV-2 test positive | 1 (14) | 0 | 1 (2) |
| General disorders, administration site conditions |  |  |  |
| Asthenia | 2 (29) | 6 (15) | 8 (17) |
| Fatigue | 0 | 8 (20) | 8 (17) |
| Pyrexia | 2 (29) | 4 (10) | 6 (13) |
| Blood and lymphatic system disorders |  |  |  |
| Anemia | 3 (43) | 10 (24) | 13 (27) |
| Neutropenia | 0 | 6 (15) | 6 (13) |
| Nervous system disorders |  |  |  |
| Neuropathy peripheral | 0 | 8 (20) | 8 (17) |
| Dysgeusia | 0 | 6 (15) | 6 (13) |
| Headache | 1 (14) | 0 | 1 (2) |
| Hypoesthesia | 1 (14) | 0 | 1 (2) |
| Musculoskeletal and connective tissue disorders |  |  |  |
| Myalgia | 0 | 7 (17) | 7 (15) |
| Arthralgia | 0 | 6 (15) | 6 (13) |
| Respiratory, thoracic, and mediastinal disorders |  |  |  |
| Dyspnea | 0 | 6 (15) | 6 (13) |
| Cough | 0 | 5 (12) | 5 (10) |
| Productive cough | 1 (14) | 1 (2) | 2 (4) |
| Vascular disorders |  |  |  |
| Hypertension | 0 | 5 (12) | 5 (10) |
| Hematoma | 1 (14) | 2 (5) | 3 (6) |
| Embolism | 1 (14) | 1 (2) | 2 (4) |
| Deep vein thrombosis | 1 (14) | 0 | 1 (2) |
| Jugular vein thrombosis | 1 (14) | 0 | 1 (2) |
| Subclavian vein thrombosis | 1 (14) | 0 | 1 (2) |
| Injury, poisoning, and procedural complications |  |  |  |
| Procedural pain | 1 (14) | 3 (7) | 4 (8) |
| Incision site pain | 1 (14) | 2 (5) | 3 (6) |
| Metabolism and nutrition disorders |  |  |  |
| Decreased appetite | 0 | 7 (17) | 7 (15) |
| Infections and infestations |  |  |  |
| COVID-19 | 2 (29) | 1 (2) | 3 (6) |
| Respiratory tract infection | 1 (14) | 1 (2) | 2 (4) |
| Sepsis | 1 (14) | 0 | 1 (2) |
| Psychiatric disorders |  |  |  |
| Insomnia | 1 (14) | 3 (7) | 4 (8) |
| Endocrine disorders |  |  |  |
| Adrenal insufficiency | 1 (14) | 0 | 1 (2) |
| Inappropriate antidiuretic hormone secretion | 1 (14) | 0 | 1 (2) |
| Ear and labyrinth disorders |  |  |  |
| Vertigo | 1 (14) | 0 | 1 (2) |
| **Adjuvant treatment period, n (%)** | Cohort A  PD-L1 high  (n = 5) | Cohort B  PD-L1 any  (n = 33) | All patients  (N = 38) |
| Gastrointestinal disorders |  |  |  |
| Constipation | 1 (20) | 5 (15) | 6 (16) |
| Nausea | 1 (20) | 5 (15) | 6 (16) |
| Diarrhea | 1 (20) | 4 (12) | 5 (13) |
| Dry mouth | 2 (40) | 3 (9) | 5 (13) |
| Lip edema | 1 (20) | 0 | 1 (3) |
| Skin and subcutaneous tissue disorders |  |  |  |
| Pruritus | 3 (60) | 5 (15) | 8 (21) |
| Rash | 0 | 6 (18) | 6 (16) |
| Rash maculo-papular | 1 (20) | 1 (3) | 2 (5) |
| Rash erythematous | 1 (20) | 0 | 1 (3) |
| Investigations |  |  |  |
| Blood creatinine increased | 2 (40) | 3 (9) | 5 (13) |
| Aspartate aminotransferase increased | 0 | 4 (12) | 4 (11) |
| Amylase increased | 1 (20) | 0 | 1 (3) |
| Blood alkaline phosphatase increased | 1 (20) | 0 | 1 (3) |
| Lipase increased | 1 (20) | 0 | 1 (3) |
| Lymphocyte count decreased | 1 (20) | 0 | 1 (3) |
| General disorders, administration site conditions |  |  |  |
| Asthenia | 2 (40) | 3 (9) | 5 (13) |
| Chest pain | 1 (20) | 1 (3) | 2 (5) |
| Mucosal inflammation | 1 (20) | 0 | 1 (3) |
| Infections and infestations |  |  |  |
| COVID-19 | 0 | 5 (15) | 5 (13) |
| Gingivitis | 1 (20) | 0 | 1 (3) |
| Respiratory tract infection | 1 (20) | 0 | 1 (3) |
| Skin infection | 1 (20) | 0 | 1 (3) |
| Metabolism and nutrition disorders |  |  |  |
| Decreased appetite | 0 | 5 (15) | 5 (13) |
| Dyslipidemia | 1 (20) | 0 | 1 (3) |
| Musculoskeletal and connective tissue disorders |  |  |  |
| Arthralgia | 2 (40) | 4 (12) | 6 (16) |
| Respiratory, thoracic, and mediastinal disorders |  |  |  |
| Cough | 0 | 4 (12) | 4 (11) |
| Pleural effusion | 1 (20) | 0 | 1 (3) |
| Rhinorrhea | 1 (20) | 0 | 1 (3) |
| Blood and lymphatic system disorders |  |  |  |
| Anemia | 2 (40) | 2 (6) | 4 (11) |
| Endocrine disorders |  |  |  |
| Hypothyroidism | 2 (40) | 2 (6) | 4 (11) |
| Vascular disorders |  |  |  |
| Hypertension | 1 (20) | 2 (6) | 3 (8) |
| Nervous system disorders |  |  |  |
| Dysgeusia | 1 (20) | 1 (3) | 2 (5) |
| Cardiac disorders |  |  |  |
| Pericardial effusion | 1 (20) | 0 | 1 (3) |
| Renal and urinary disorders |  |  |  |
| Hematuria | 1 (20) | 0 | 1 (3) |

AEs, adverse events; PD-L1, programmed death-ligand; MedDRA: Medical Dictionary for Regulatory Activities.

**Supplementary Table 3.** AESIs during the neoadjuvant and adjuvant treatment periods.

| **Neoadjuvant treatment period, n (%)** | Cohort A  PD-L1 high  (n = 7) | Cohort B  PD-L1 any (n = 41) | All patients  (N = 48) |
| --- | --- | --- | --- |
| Patients with at least one AESI | 5 (71) | 28 (68) | 33 (69) |
| Total number of AESIs | 12 | 48 | 60 |
| Patients with at least one of the following: |  |  |  |
| Treatment-related AESI |  |  |  |
| Any treatment | 5 (71) | 24 (59) | 29 (60) |
| Tiragolumab | 5 (71) | 20 (49) | 25 (52) |
| Atezolizumab | 4 (57) | 21 (51) | 25 (52) |
| Chemotherapy | 0 | 6 (15) | 6 (13) |
| Grade 3/4 AESI | 2 (29) | 2 (5) | 4 (8) |
| Treatment-related Grade 3/4 AESI | 2 (29) | 2 (5) | 4 (8) |
| Grade 5 AESI | 0 | 0 | 0 |
| Treatment-related Grade 5 AESI | 0 | 0 | 0 |
| Serious AESI | 1 (14) | 0 | 1 (2) |
| Treatment-related serious AESI | 1 (14) | 0 | 1 (2) |
| AESI requiring corticosteroid use | 1 (14) | 12 (29) | 13 (27) |
| AESI requiring immunosuppressant other than corticosteroid | 1 (14) | 0 | 1 (2) |
| AESI leading to withdrawal from any treatment | 0 | 0 | 0 |
| AESI leading to any dose modification or interruption |  |  |  |
| Any treatment | 0 | 2 (5) | 2 (4) |
| Tiragolumab | 0 | 2 (5) | 2 (4) |
| Atezolizumab | 0 | 1 (2) | 1 (2) |
| Chemotherapy | 0 | 1 (2) | 1 (2) |
| Immune-mediated AESIs |  |  |  |
| Rash | 4 (57) | 17 (42) | 21 (44) |
| Hepatitis (diagnosis and lab abnormalities) | 3 (43) | 9 (22) | 12 (25) |
| Hepatitis (lab abnormalities) | 3 (43) | 9 (22) | 12 (25) |
| Hypothyroidism | 0 | 4 (10) | 4 (8) |
| Infusion-related reactions | 0 | 3 (7) | 3 (6) |
| Adrenal insufficiency | 1 (14) | 0 | 1 (2) |
| Hyperthyroidism | 0 | 1 (2) | 1 (2) |
| Severe cutaneous reactions | 0 | 1 (2) | 1 (2) |
| **Adjuvant treatment period, n (%)** | Cohort A  PD-L1 high  (n = 5) | Cohort A  PD-L1 all (n = 33) | All patients  (N = 38) |
| Patients with at least one AESI | 2 (40) | 20 (61) | 22 (58) |
| Total number of AESIs | 8 | 35 | 43 |
| Patients with at least one of the following: |  |  |  |
| Treatment-related AESI |  |  |  |
| Any treatment | 2 (40) | 17 (52) | 19 (50) |
| Tiragolumab | 2 (40) | 16 (49) | 18 (47) |
| Atezolizumab | 2 (40) | 17 (52) | 19 (50) |
| Chemotherapy | 0 | 1 (3) | 1 (3) |
| Grade 3/4 AESI | 0 | 5 (15) | 5 (13) |
| Treatment-related Grade 3/4 AESI | 0 | 4 (12) | 4 (11) |
| Grade 5 AESI | 0 | 0 | 0 |
| Treatment-related Grade 5 AESI | 0 | 0 | 0 |
| Serious AESI | 0 | 2 (6) | 2 (5) |
| Treatment-related serious AESI | 0 | 2 (6) | 2 (5) |
| AESI requiring corticosteroid use | 1 (20) | 7 (21) | 8 (21) |
| AESI requiring immunosuppressant other than corticosteroid | 0 | 1 (3) | 1 (3) |
| AESI leading to withdrawal from any treatment |  |  |  |
| Any treatment | 0 | 8 (24) | 8 (21) |
| Tiragolumab | 0 | 8 (24) | 8 (21) |
| Atezolizumab | 0 | 8 (24) | 8 (21) |
| Chemotherapy | 0 | 0 | 0 |
| AESI leading to any dose modification or interruption |  |  |  |
| Any treatment | 1 (20) | 5 (15) | 6 (16) |
| Tiragolumab | 1 (20) | 5 (15) | 6 (16) |
| Atezolizumab | 1 (20) | 5 (15) | 6 (16) |
| Chemotherapy | 0 | 0 | 0 |
| Immune-mediated AESIs |  |  |  |
| Rash | 1 (20) | 9 (27) | 10 (26) |
| Hepatitis (diagnosis and lab abnormalities) | 0 | 7 (21) | 7 (18) |
| Hypothyroidism | 2 (40) | 5 (15) | 7 (18) |
| Hepatitis (lab abnormalities) | 0 | 5 (15) | 5 (13) |
| Pneumonitis | 0 | 3 (9) | 3 (8) |
| Colitis | 0 | 2 (6) | 2 (5) |
| Hepatitis (diagnosis) | 0 | 2 (6) | 2 (5) |
| Myositis | 0 | 2 (6) | 2 (5) |
| Myositis (myositis and rhabdomyolysis) | 0 | 2 (6) | 2 (5) |
| Adrenal insufficiency | 0 | 1 (3) | 1 (3) |
| Ocular inflammatory toxicity | 0 | 1 (3) | 1 (3) |
| Pancreatitis | 1 (20) | 0 | 1 (3) |

AESIs, adverse events of special interest; PD-L1, programmed death-ligand.

**Supplementary Table 4.** Summary of best overall response by investigator during the neoadjuvant phase

| **n (%)**  **(95% CI)** | Cohort A  PD-L1 high  (n = 7) | Cohort B  PD-L1 any  (n = 41) | All patients  (N = 48) |
| --- | --- | --- | --- |
| Responders | 4 (57)  (20, 88) | 27 (66)  (49, 79) | 31 (65)  (49, 77) |
| CR | 1 (14)  (1, 58) | 1 (2)  (0, 14) | 2 (4)  (1, 15) |
| PR | 3 (43)  (12, 80) | 26 (63)  (47, 77) | 29 (60)  (45, 74) |
| SD | 2 (29)  (5, 70) | 13 (32)  (19, 48) | 15 (31)  (19, 46) |
| PD | 1 (14)  (1, 58) | 0  (0, 11) | 1 (2)  (0, 12) |
| NE | 0 | 0 | 0 |
| Missing | 0 | 1 (2) | 1 (2) |

CR, compete response; NE, not evaluable; PD, disease progression; PR, partial response; SD, stable disease

**Supplementary Figure 1.** Role functioning in Cohort B.

*Scores for role functioning were calculated based on patients' ability to perform their work, daily activities, hobbies, and leisure activities. The scale is scored from 0 to 100, with a higher score indicating better role functioning.

C, cycle; D, day; PD-L1, programmed death-ligand 1.

**Supplementary Figure 2.** Physical functioning in Cohort B.

 C, cycle; D, day; EORTC-QLQ-C30, European Organisation for Research and Treatment of Cancer Quality of Life Questionnaire–Core 3, PD-L1, programmed death-ligand 1.
